# Synthesis, Characterization, and Antifungal Property of Hydroxypropyltrimethyl Ammonium Chitosan Halogenated Acetates

**DOI:** 10.3390/md16090315

**Published:** 2018-09-05

**Authors:** Yingqi Mi, Wenqiang Tan, Jingjing Zhang, Lijie Wei, Yuan Chen, Qing Li, Fang Dong, Zhanyong Guo

**Affiliations:** 1 Key Laboratory of Coastal Biology and Bioresource Utilization, Yantai Institute of Coastal Zone Research, Chinese Academy of Sciences, Yantai 264003, China; yqmi@yic.ac.cn (Y.M.); wqtan@yic.ac.cn (W.T.); jingjingzhang@yic.ac.cn (J.Z.); ljwei@yic.ac.cn (L.W.); yuanchen@yic.ac.cn (Y.C.); qli@yic.ac.cn (Q.L.); 2 University of Chinese Academy of Sciences, Beijing 100049, China

**Keywords:** hydroxypropyltrimethyl ammonium chloride chitosan, halogenated acetate, antifungal activity, electronegativity

## Abstract

Hydroxypropyltrimethyl ammonium chitosan halogenated acetates were successfully synthesized from six different haloacetic acids and hydroxypropyltrimethyl ammonium chloride chitosan (HACC) with high substitution degree, which are hydroxypropyltrimethyl ammonium chitosan bromacetate (HACBA), hydroxypropyltrimethyl ammonium chitosan chloroacetate (HACCA), hydroxypropyltrimethyl ammonium chitosan dichloroacetate (HACDCA), hydroxypropyltrimethyl ammonium chitosan trichloroacetate (HACTCA), hydroxypropyltrimethyl ammonium chitosan difluoroacetate (HACDFA), and hydroxypropyltrimethyl ammonium chitosan trifluoroacetate (HACTFA). These chitosan derivatives were synthesized by two steps: first, the hydroxypropyltrimethyl ammonium chloride chitosan was synthesized by chitosan and 3-chloro-2-hydroxypropyltrimethyl ammonium chloride. Then, hydroxypropyltrimethyl ammonium chitosan halogenated acetates were synthesized via ion exchange. The structures of chitosan derivatives were characterized by Fourier transform infrared spectroscopy (FTIR), ^1^H Nuclear magnetic resonance spectrometer (^1^H NMR), ^13^C Nuclear magnetic resonance spectrometer (^13^C NMR), and elemental analysis. Their antifungal activities against *Colletotrichum lagenarium*, *Fusarium graminearum*, *Botrytis cinerea*, and *Phomopsis asparagi* were investigated by hypha measurement in vitro. The results revealed that hydroxypropyltrimethyl ammonium chitosan halogenated acetates had better antifungal activities than chitosan and HACC. In particular, the inhibitory activity decreased in the order: HACTFA > HACDFA > HACTCA > HACDCA > HACCA > HACBA > HACC > chitosan, which was consistent with the electron-withdrawing property of different halogenated acetates. This experiment provides a potential idea for the preparation of new antifungal drugs by chitosan.

## 1. Introduction

Agricultural diseases resulted from plant pathogenic fungi may cause the large crop death, which limits crop production worldwide, and can lead to great financial losses to farmers [1,2]. There are various kinds of plant pathogenic fungi, with various modes of action. For instance, fusarium wilt in watermelon caused by *Fusarium oxysporum* (*F. oxysporum*) is a common disease in several countries including the China and United States, which can lead to massive loss to watermelon production [3]. *Gibberella zeae* (*G. zeae*) distributed all over the world is a devastating filamentous fungus. It can cause fusarium head blight disease in several economically important crops, such as maize, barley, and wheat [4,5]. How to prevent these diseases caused by plant pathogenic fungi is a thought-provoking question. Currently, the most common way to control the plant fungous diseases is to use massive chemical fungicides [6,7]. However, due to the excessive use of the chemical fungicides, the ecological environment has been seriously damaged [8]. So, it is urgent to research a kind of new drug with low toxicity and strong antifungal activity.

There are many bioactive compounds in the marine which can translate into fungicide alternatives [2]. Chitosan prepared by the *N*-deacetylation of chitin is one of the active substances in the ocean [9]. Chitosan, as a nontoxic and renewable natural polysaccharide, has good biocompatibility and biodegradability. In addition, it also has the characteristics of film-forming ability, low toxicity, antibacterial activity, and so on [10,11]. Therefore, chitosan has been drawing extensive attention in agriculture, medicine, food science, industry, and environmental protection due to its unique physical and chemical properties in recent years. However, compared with current fungicides, its relatively low antibacterial activity and poor solubility inhibit its feasibility as a kind of fungicide [12,13]. So, chemical modification of chitosan in order to obtain various chitosan derivatives which are active and water-soluble is the focus of research.

The structural modification sites of chitosan are mainly C_2_-NH_2_ and C_6_-OH [14]. For instance, the C_2_-NH_2_ of chitosan can react with aldehydes or ketones to form the corresponding aldimines and ketimines, which is called Schiff base, and *O*-carboxymethyl chitosan can be formed by the introduction of carboxyl groups on the C_6_-OH [15,16,17]. In general, chitosan derivatives with different activities can be synthesized through chemical modification such as acylation, alkylation, Schiff base reaction, quaternary ammonium reaction and so on. Among the above chemical modification, chitosan quaternary ammonium salt has attracted people’s attention for its unique character. As one of the kind of chitosan quaternary ammonium salt, hydroxypropyltrimethyl ammonium chloride chitosan (HACC) is a polycationic compound, characterized by good water solubility, flocculation, moisture absorption, and antibacterial properties with low cytotoxicity to cells [11]. At the same time, HACC can be prepared by simple method with high synthesis efficiency and little harm to the environment. In recent years, many studies showed that HACC had potential applications in many fields. For instance, HACC has been reported as a chitosan derivative with enhanced antibacterial ability against *Escherichia coli*, *Candida albicans*, and *Staphylococcus aureus* [18]. And the antibacterial drugs made by HACC have been widely used. Meanwhile, it is reported that HACC can be used in other areas, such as nanofiltration, orthopedics, and drug delivery, due to its water solubility, low cytotoxicity to cells, and biocompatibility [19]. For example, HACC possesses the stronger electrostatic interaction with negatively charged tumor cells when it is used as a drug carrier for cancer treatment [20]. However, the antifungal activity of HACC is not optimistic according to the earlier studies [18]. Furthermore, it is reported that the halogens have good antifungal activity. It is assumed that the electron-withdrawing substitution—halogens can play a crucial part in the antifungal properties of compounds, which can destroy cell walls and membranes to lead to the death of fungus [21,22]. Chemical fungicides with halogens are widely used in recent years due to its effective antifungal activity. However, the chemical fungicides cause serious problems for the environment because of toxicities and residues. When the halogens are grafted to chitosan, they should be released slowly and may meet requirements of environmental safety.

On the basis of the above statement, we modified hydroxypropyltrimethyl ammonium chitosan via six different haloacetic acids to obtain high antifungal activity and water-soluble chitosan derivatives. Firstly, the hydroxypropyltrimethyl ammonium chloride chitosan was synthesized by chitosan and 3-chloro-2-hydroxypropyltrimethyl ammonium chloride. Then, hydroxypropyltrimethyl ammonium chitosan halogenated acetates were synthesized via ion exchange. The structures of chitosan derivatives were characterized by Fourier transform infrared spectroscopy (FTIR), ^1^H Nuclear magnetic resonance spectrometer (^1^H NMR), ^13^C Nuclear magnetic resonance spectrometer (^13^C NMR), and elemental analyses. Their antifungal activities against *Colletotrichum lagenarium* (*C. lagenarium*), *Fusarium oxysporum* (*F. oxysporum*), *Botrytis cinerea* (*B. cinerea*), and *Phomopsis asparagi* (*P. asparagi*) were investigated by hypha measurement in vitro. Based on the obtained data, the relationship between chitosan derivatives and antifungal activity was discussed briefly.

## 2. Results and Discussion

### 2.1. Chemical Synthesis and Characterization

**Scheme 1:**
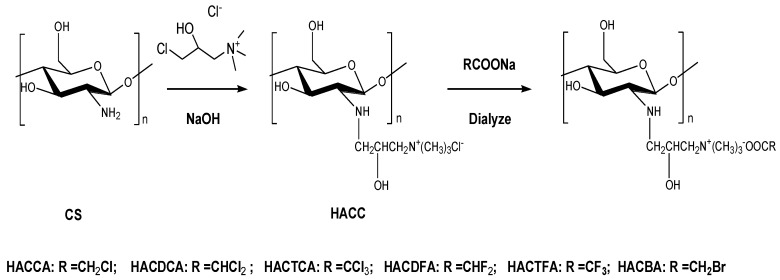
Synthesis routes for chitosan derivatives.

**Figure 1:**
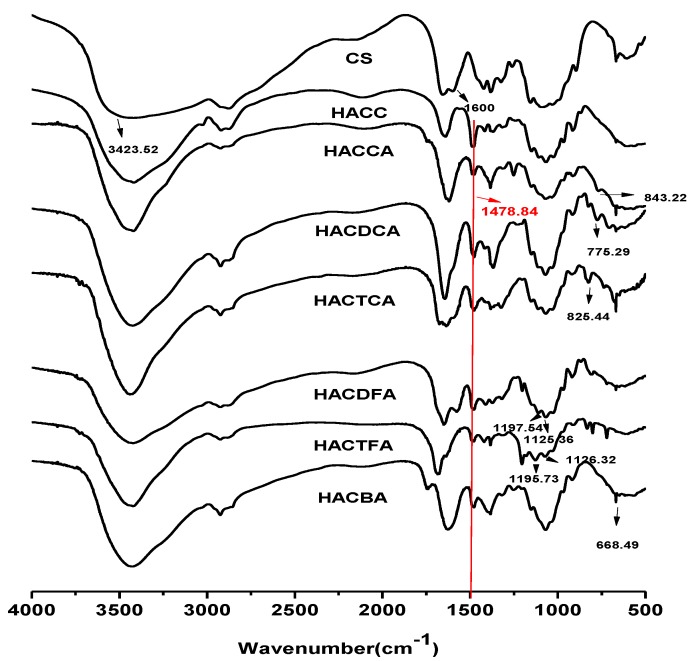
Fourier transform infrared spectroscopy (FTIR) spectra of chitosan and chitosan derivatives.

**Figure 2:**
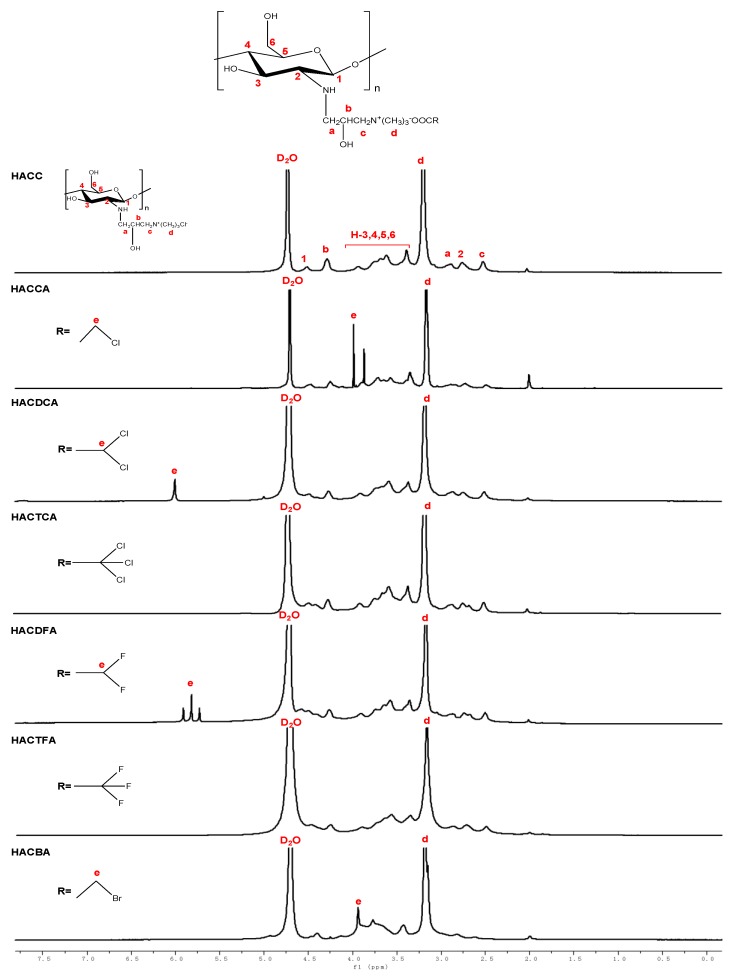
^1^H Nuclear magnetic resonance spectrometer (^1^H NMR) spectra of chitosan derivatives.

**Figure 3:**
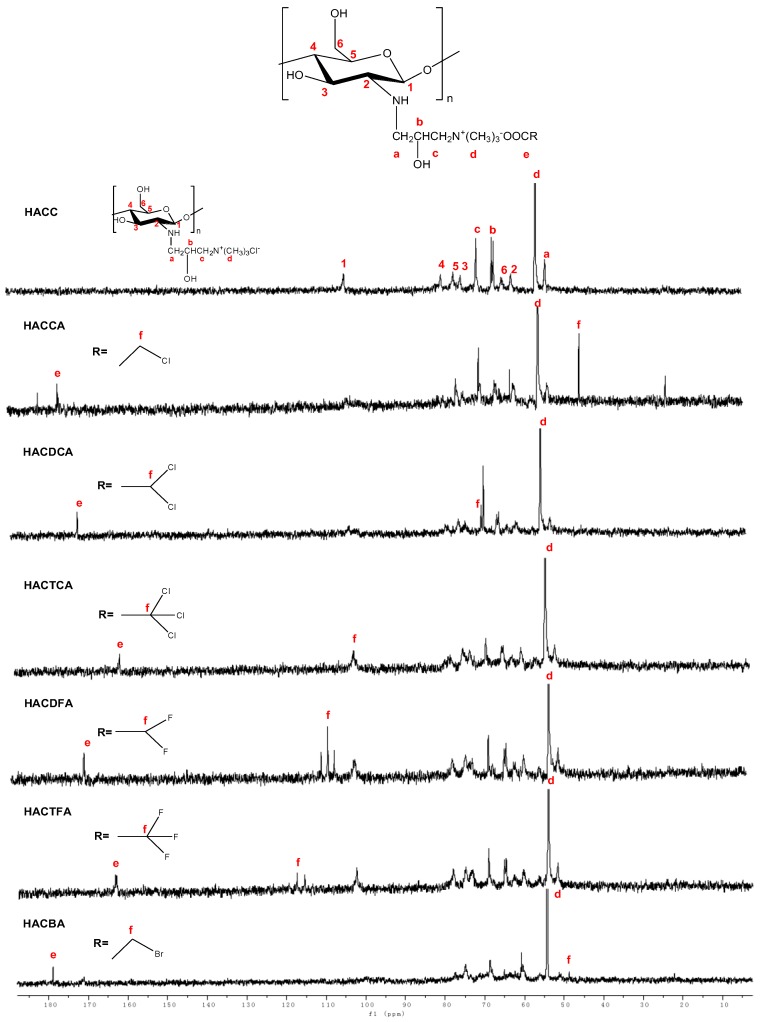
^13^C Nuclear magnetic resonance spectrometer (^13^C NMR) spectra of chitosan derivatives.

The synthetic strategy of hydroxypropyltrimethyl ammonium chitosan halogenated acetates is shown in Scheme 1. Firstly, hydroxypropyltrimethyl ammonium chloride chitosan (HACC) was prepared by chitosan and 3-chloro-2-hydroxypropyltrimethyl ammonium chloride. Then, HACC was dissolved in 20% sodium halogenated acetate for purpose of replacing the chloride ions with haloacetic ions. Hydroxypropyltrimethyl ammonium chitosan halogenated acetates were obtained after drying in vacuo. The structures of chitosan derivatives were characterized by FTIR (Figure 1), ^1^H NMR (Figure 2), ^13^C NMR (Figure 3), and elemental analysis (Table 1), respectively.

#### 2.1.1. Fourier Transform Infrared Spectroscopy (FTIR) Spectra

Figure 1 shows the spectra of chitosan, intermediate product, and chitosan derivatives respectively. For chitosan, the peaks appear at 3423.62, 2922.51, 1650.24, 1600.21, and 1072.24 cm^−1^ [23]. The peak of 3423.62 cm^−1^ presents the characteristic absorbance of -OH and –NH_2_. The weak peak at 2922.51 cm^−1^ shows the stretching vibration of –CH [24,25]. The band at 1650.24 cm^−1^ can be attributed to C=O (the amide I) stretching vibration. The band at 1600.21 cm^−1^ can be attributed to –NH_2_. The peak at 1072.24 cm^−1^ shows the stretching vibrations of the C–O [26,27]. The IR spectrum of HACC (Figure 1) shows the introduction of the quaternary ammonium salt group to the chitosan backbone. Compared to chitosan, a peak at 1478.84 cm^−1^ is attributed to the C-H bending of the trimethylammonium group [18], and a peak at 1600.21 cm^−1^ weakens greatly for the partial change of the primary amine of chitosan to the secondary amine. For HACCA, HACDCA, and HACTCA, new peaks appeared at 843.22, 776.28, 826.44 cm^−1^ respectively can be attributed to the C-Cl bending [28,29]. For HACDFA, new peaks appear at 1187.64 and 1126.38 cm^−1^ for the C-F bending. Meanwhile, new peaks appear at 1186.73 and 1128.32 cm^−1^ are assigned to the characteristic absorbance of C-F for HACTFA [10]. For HACBA, a new peak appears at 668.49 cm^−1^, which can be attributed to the C-Br bending [25,30]. In addition, the peak at 1478.84 cm^−1^ attributed to the C–H bending of the trimethylammonium group still exists in the molecules of HACCA, HACDCA, HACTCA, HACDFA, HACTFA, and HACBA.

#### 2.1.2. Nuclear Magnetic Resonance Spectrometer (NMR) Spectra

^1^H NMR spectra of intermediate products HACC and chitosan derivatives are shown in Figure 2. As shown in the figure, chemical shifts at 4.47 ppm, 3.24–4.02 ppm, and 2.72 ppm are assigned to [H1], [H3]–[H6], and [H2] of chitosan [31]. In ^1^H NMR spectrum of HACC, an obvious characteristic peak of hydrogen (3.16 ppm) of trimethyl ammonium groups is appeared [18]. At the same time, for HACC, the other signals can be well observed: *δ* = 2.41 ppm attributed to –CH_2_, *δ* = 2.69 ppm (a), *δ* = 4.50 ppm (b) [21,24,32]. For HACCA, compared to HACC, a new peak appears at 4.21 ppm, which can be assigned to protons of halogenated acetic anions (e). Furthermore, for HACDCA, HACDFA, HACBA, new peaks appear at 6.12 ppm, 5.75–6.01 ppm, and 3.78 ppm, which can be attributed to protons of halogenated acetic anions (e) [10,29]. However, the spectra of HACTCA and HACTFA are similar to HACC, and no new peaks appear because of the lack of protons of halogenated acetic anions. In addition, the chemical shifts of trimethyl ammonium groups at about 3.16 ppm still exist in the spectra of HACCA, HACDCA, HACTCA, HACDFA, HACTFA, and HACBA.

^13^C NMR spectra of intermediate products HACC and chitosan derivatives are shown in Figure 3. As shown in the figures, all spectra show signals at 55.10–105.68 ppm (^13^C NMR spectra) [26], which are assigned to the diagnostic chemical shifts of chitosan. In ^13^C NMR spectrum of HACC, an obvious characteristic peak of carbon (57.25 ppm) of trimethyl ammonium groups is appeared [18]. Meanwhile, for HACC, the other signals can be well observed: *δ* = 51.85 ppm attributed to –CH_2_, *δ* = 64.81 ppm (b), *δ* = 69.19 ppm (c) [29]. For hydroxypropyltrimethyl ammonium chitosan halogenated acetates, compared to HACC, new peaks appear at 174.83, 170.80, 161.41, 169.86, 162.93, and 178.90 ppm, which can be assigned to carbons of COO^−^ groups in HACCA, HACDCA, HACTCA, HACDFA, HACTFA, and HACBA (e) [10,28]. Furthermore, for hydroxypropyltrimethyl ammonium chitosan halogenated acetates, new peaks appear at 43.91 ppm (CH_2_Cl in HACCA), 69.47 ppm (CHCl_2_ in HACDCA), 102.47 ppm (CCl_3_ in HACTCA), 109.14 ppm (CHF_2_ in HACDFA), 117.35 ppm (CF_3_ in HACTFA), and 48.78 ppm (CH_2_Br in HACBA), which can confirm the presence of the halogenated methyl carbons [10,26,28]. In addition, the chemical shifts of trimethyl ammonium groups at about 57.25 ppm still exist in the spectra of HACCA, HACDCA, HACTCA, HACDFA, HACTFA, and HACBA.

These data indicate that hydroxypropyltrimethyl ammonium chitosan halogenated acetates are successfully synthesized. And according to the ^1^H NMR spectra, the degrees of substitution of chitosan derivatives were calculated by using the integration of [H2] as an integral standard peak [20]. The degrees of substitution of HACC, HACCA, HACDCA, HACDFA, and HACTFA was determined as 71.30, 66.00%, 61.00%, 48.00%, and 68.00%, respectively (Table S1). However, the degrees of substitution of HACTCA and HACTFA can not calculate by the ^1^H NMR spectra. Therefore, we did the elemental analysis in order to calculate the degrees of substitution.

#### 2.1.3. Elemental Analysis

The yields and the degrees of substitution of chitosan derivatives are shown in Table 1. The changes in W_C/N_ indicate the functional groups grafting on chitosan successfully. From the data in Table 1, the intermediate HACC presents the highest degree of substitution. The degrees of substitution of the other six chitosan derivatives are different. HACTCA, for example, has a far lower degree of substitution than HACDFA, which is 48.64%.

### 2.2. Antifungal Activity

**Figure 4:**
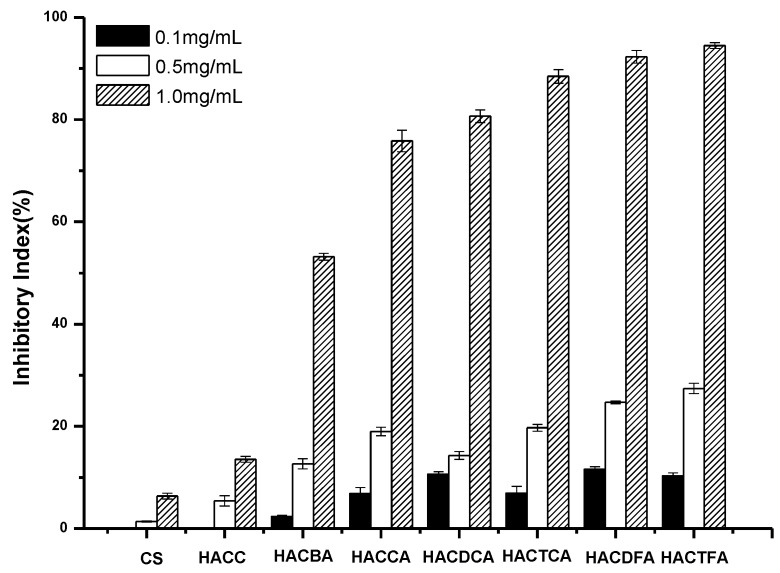
The antifungal activity of chitosan and chitosan derivatives against *Colletotrichum lagenarium*.

**Figure 5:**
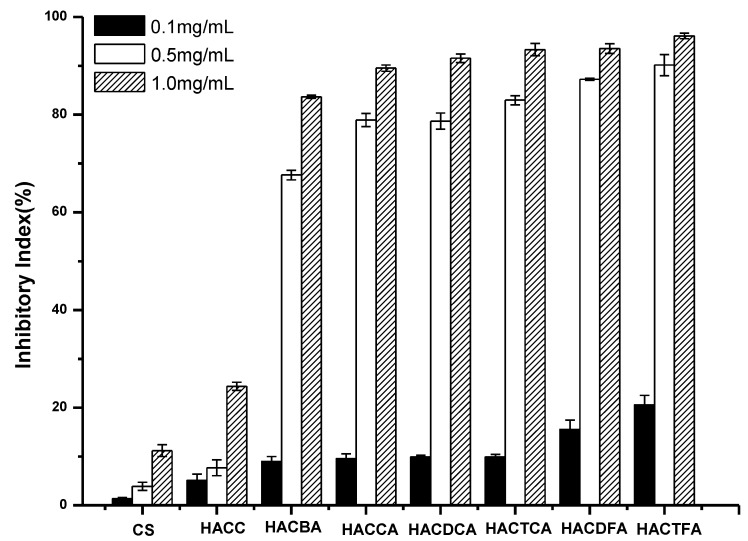
The antifungal activity of chitosan and chitosan derivatives against *Fusarium oxysporum*.

**Figure 6:**
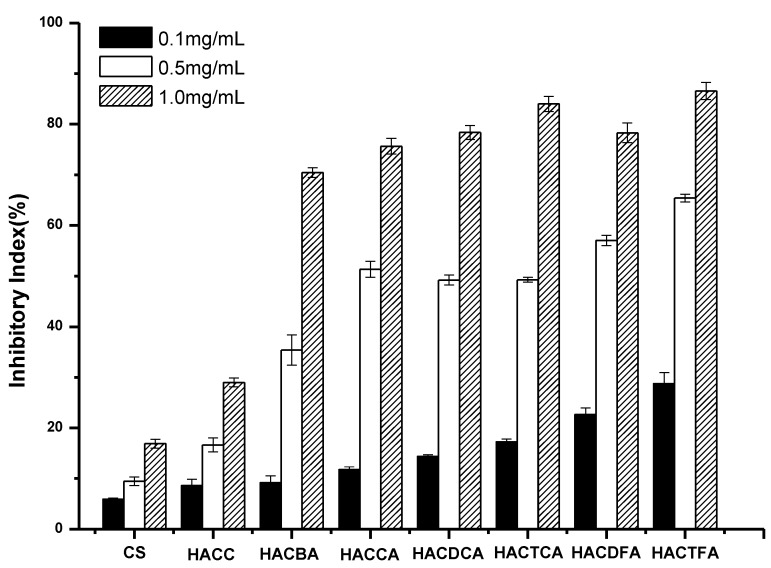
The antifungal activity of chitosan and chitosan derivatives against *Botrytis cinerea*.

**Figure 7:**
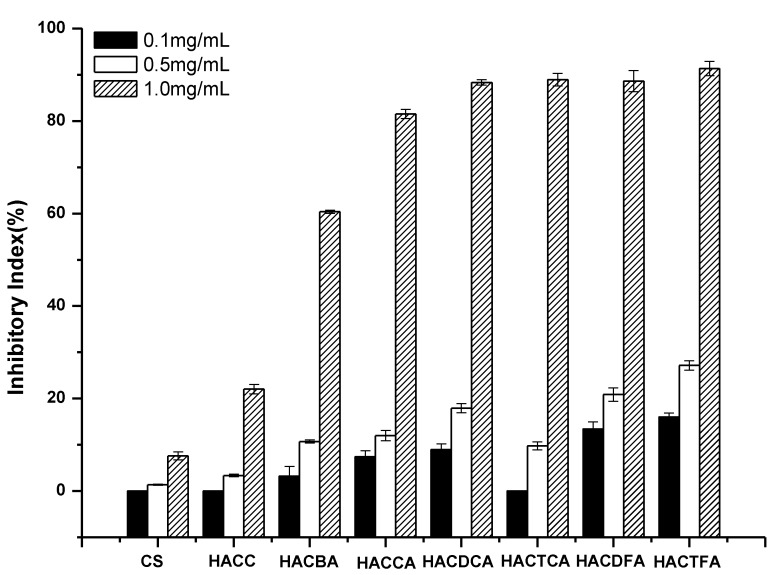
The antifungal activity of chitosan and chitosan derivatives against *Phomopsis asparagi*.

Antifungal assays against *C. lagenarium*, *F. oxysporum*, *B. cinerea*, and *P. asparagi* are performed by following the plate growth rate method. The antifungal activity of chitosan and its derivatives is shown in Figure 4, Figure 5, Figure 6 and Figure 7.

*C. lagenarium*, which is caused by the introduction of pathogenic bacteria in the seeds, has been increasingly harmful in recent years. It has a great impact on crop production, and is difficult to control. Figure 4 shows the antifungal activities of chitosan, intermediates, and target products against *C. lagenarium*. Chitosan and HACC have almost no inhibitory effect on *C. lagenarium*. However, the enhanced antifungal activities of the target products including HACCA, HACDCA, HACTCA, HACDFA, HACTFA, and HACBA are very obvious. Specifically, the conclusions are as follows: firstly, all the target products including HACCA, HACDCA, HACTCA, HACDFA, HACTFA, and HACBA have much stronger antifungal activity. Secondly, with the augment of sample concentration, the antifungal activity of the target product increases gradually. For example, when the concentrations of HACTFA are 0.1 mg/mL, 0.5 mg/mL, and 1.0 mg/mL, the inhibition rates on *C. lagenarium* are 17.14%, 30.09%, and 96.57% respectively. Thirdly, the order of inhibition of *C. lagenarium* is: HACTFA > HACDFA > HACTCA > HACDCA > HACCA > HACBA. Meanwhile, it is clear that the inhibition rate of HACDFA with DS of 48.64% is 5.64 percent higher than that of HACDCA with degree of substitution of 56.23% at 1.0 mg/mL, which can reasonably suggest that higher inhibitory index of HACDFA should be possible if they have the same degree of substitution. Similarly, HACCA has better antifungal activity than HACBA, despite its low degree of substitution. Therefore, the expected order of inhibition should be as follows: HACTFA > HACDFA > HACTCA > HACDCA > HACCA > HACBA, which is identical to the experimental results. And this rule is consistent with the order of the electronegativity of halogen-containing substituents in the hydroxypropyltrimethyl ammonium chitosan halogenated acetates.

*F. oxysporum* is a common disease in several countries, which can lead to massive loss to watermelon production. Figure 5 shows the antifungal activities against *F. oxysporum* of chitosan and chitosan derivatives. Compared to chitosan and HACC, the antifungal activity against *F. oxysporum* of HACCA, HACDCA, HACTCA, HACDFA, HACTFA, and HACBA have been greatly improved. Specifically, antifungal activity increases with the augment of sample concentration. When the sample concentration is 1.0 mg/mL, and compared to chitosan with the inhibitory index 13.21%, the inhibitory indices of HACCA, HACDCA, HACTCA, HACDFA, HACTFA, and HACBA are 92.81%, 93.40%, 93.87%, 94.54%, 94.97%, and 97.50%, respectively. Meanwhile, after the introduction of active groups, chitosan derivatives have a particularly well antifungal effect on *F. oxysporum*, which inhibitory index reaches above 70.52% at the sample concentration of 0.5 mg/mL. Considering the relationships between degree of substitution and inhibitory index, the antifungal activities of chitosan derivatives are ranked as follows: HACTFA > HACDFA > HACTCA > HACDCA > HACCA > HACBA.

As shown in Figure 6, chitosan and HACC possess little antifungal activity. However, hydroxypropyltrimethyl ammonium chitosan halogenated acetates have antifungal activity at all the tested concentration against *B. cinerea*. The inhibitory indices of all the samples mount up with the increasing concentration. After the introduction of active groups, the antifungal activity of all the target products is better than chitosan raw materials and intermediate product. Especially, this trend is apparently reinforced at 1.0 mg/mL, and compared to the inhibitory index of chitosan and HACC, the inhibitory indices of HACCA, HACDCA, HACTCA, HACDFA, HACTFA, and HACBA are 75.64%, 78.35%, 84.02%, 78.29%, 86.57%, and 70.46%, respectively. As previously described, the order of antifungal activity is HACTCA > HACDCA > HACCA. However, contrary to the previous description, the inhibitory property of HACDFA is slightly lower than HACTCA, which is possibly because of its lower substitution degree (Table 1) and less active ingredient.

Asparagus stem blight is a highly destructive disease of asparagus in China and some Asian countries. Due to the filamentous fungus, *P. asparagi* is identified as the causing pathogen, it is particularly important to inhibit the activity of *P. asparagi*. As shown in Figure 7, apart from HACC, chitosan derivatives have outstanding antifungal activities at all the tested concentration against *P. asparagi*. The inhibitory indices of all the samples mount up with increasing concentration. All chitosan derivatives show stronger antifungal activity than chitosan and HACC. Especially, this trend reinforces apparently at 1.0 mg/mL, and compared to chitosan with the inhibitory index 7.59%, the inhibitory indices of HACBA, HACCA, HACDCA, HACTCA, HACDFA, and HACTFA are 60.47%, 81.58%, 88.42%, 89.05%, 88.69, and 90.45%, respectively. In summary, except for HACDFA and HACTCA, the antifungal activities are approximate, and the other antifungal activities are consistent with the previous description.

The results showed that the six different kinds of chitosan derivatives including HACCC, HACCDC, HACCTC, HACCDF, HACCTF, and HACCB, had much stronger inhibitory effect on *C. lagenarium*, *F. oxysporum*, *B. cinerea*, and *P. asparagi*. In a nutshell, the antifungal activity decreased in the order: HACTFA > HACDFA > HACTCA > HACDCA > HACCA > HACBA > HACC > chitosan. To be specific, the conclusions was as follows: firstly, all the target products including HACCA, HACDCA, HACTCA, HACDFA, HACTFA, and HACBA had much stronger antifungal activities on *C. lagenarium*, *F. oxysporum*, *B. cinerea*, and *P. asparagi*. The results confirmed that the introduction of halogen greatly enhanced the antifungal activity of chitosan derivatives. Because the halogen groups could inhibit synthesis of the cell membrane and cell wall to exhibit antifungal activity [24]. Specifically speaking, the halogen atoms, which had a high electron-withdrawing property, could increase the hydrophobicity of chitosan derivatives. The structures of the outer membranes as well as the internal membranes of microbial cells were impacted by the changes in hydrophobicity, and could prevent nutrient substances from entering cells [26]. The microorganisms would die for these reasons. Secondly, the antifungal activity of chitosan derivatives varies with the types and quantities of halogen atoms. On one hand, HACCA, HACDCA, HACTCA had the same types of halogen-Cl, and they exhibited different antifungal activities due to the different amounts of halogen groups. That was to say the capacity of electron-withdrawing would enhance with the amounts of halogen groups. So, the antifungal activities of the target products showed the order of HACTCA > HACDCA > HACCA. On the other hand, HACTFA and HACTCA, which had the same amount of halogens, demonstrated different antifungal activities. HACTFA showed stronger antifungal activity compared with HACTCA, which could be attributed to the higher electronegativity of fluorine atoms than chlorine atoms. Therefore, the antifungal potential of target products was related to the electronegativity of halogen substituents [11,30,31,32]. In short, the higher degree of electron-withdrawing property the halogen substituents possessed, the higher the positive charge density of cationic amino possessed. As a result, the chitosan derivatives showed stronger antifungal activity [33,34,35]; this was consistent with our experimental results.

## 3. Materials and Methods

### 3.1. Materials

Chitosan was purchased from Qingdao Baicheng Biochemical Corp. (Qingdao, China). Its degree of deacetylation was 85%, and the viscosity-average molecular weight was 2.0 × 10^5^. 3-chloro-2-hydroxypropyltrimethyl ammonium chloride, isopropanol, chloroacetic acid, dichloroacetic acid, trichloroacetic acid, difluoroacetic acid, trifluoroacetic acid, and bromoacetic acid were purchased from Sigma-Aldrich Chemical Corp. (Shanghai, China). Ethanol and sodium hydroxide were all purchased from Sinopharm Chemical Reagent Co., Ltd. (Shanghai, China). The reagents were analytical grade and used without further purification. Fungi medium was purchased from Qingdao Hop Bio-Technogy Co., Ltd. (Qingdao, China). Agar powder was purchased from Beijing Chembase Technology Co., Ltd. (Beijing, China).

### 3.2. Analytical Methods

#### 3.2.1. Fourier Transform Infrared (FTIR) Spectroscopy

FTIR spectra were performed ranging from 4000 cm^−1^ to 400 cm^−1^ using a Jasco-4100 (Tokyo, Japan), provided by JASCO China (Shanghai) Co. Ltd. (Shanghai, China). All samples were ground and mixed with KBr disks for testing.

#### 3.2.2. Nuclear Magnetic Resonance (NMR) Spectroscopy

^1^H NMR spectra and ^13^C NMR spectra were recorded on samples dissolved in D_2_O with a Bruker AVIII-500 Spectrometer (500 MHz, Fällanden, Switzerland, provided by Bruker Tech and Serv. Co., Ltd., Beijing, China) at 25 °C.

#### 3.2.3. Elemental Analysis

The elemental analyses (C, H, and N) were performed on a Vario Micro Elemental Analyzer (Elementar, Hanau, Germany), and they can be used to evaluate the degree of substitution of chitosan derivatives. The degrees of substitution (DS) of chitosan derivatives were calculated according to the carbon nitrogen ratio, and the formula as follows [10,26]:

(1) $$DS_{1}=\frac{n_{1}*M_{C}-M_{N}*W_{C/N}}{n_{2}*M_{C}}\;$$

(2) $$DS_{2}=\frac{n_{1}*M_{C}-n_{2}*M_{C}*DS_{1}-M_{N}*W_{C/N}}{M_{N}*W_{C/N}-n_{3}*M_{C}}\;$$

(3) $$DS_{3}=\frac{M_{N}*W_{C/N}+M_{N}*DS_{2}*W_{C/N}-n_{1}*M_{C}*DS_{2}-n_{2}*M_{C}*DS_{1}}{n_{4}*M_{C}}\;$$

where *DS*_1_, *DS*_2_, *DS*_3_ represent the deacetylation degree of chitosan, the degrees of substitution of HACC, and degrees of substitution of hydroxypropyltrimethyl ammonium chitosan halogenated acetates; *M*_C_ and *M*_N_ are the molar masses of carbon and nitrogen, *M_C_* = 12, *M*_N_ = 14, respectively; *n*_1_, *n*_2_, *n*_3_ *n*_4_ are the number of carbons of chitin, acetamido group, 2-chloromethyl ammonium chloride, and haloacetic acid group, *n*_1_ = 8, *n*_2_ = 2, *n*_3_ = 6, *n*_4_ = 2, respectively; *W*_C/N_ represents the mass ratio between carbon and nitrogen in chitosan derivatives.

### 3.3. Synthesis of Chitosan Derivatives

#### 3.3.1. Synthesis of Hydroxypropyltrimethyl Ammonium Chloride Chitosan (HACC)

The reaction scheme for the synthesis of HACC is shown in scheme 1. HACC was prepared as follows: chitosan (5 g) was dissolved in 20 mL isopropanol prior to the addition of 9 mL 40% (*w*/*v*) NaOH aqueous solution, and stirred at 60 °C for 4 h. Then, 20 mL 60% (*w*/*v*) hydroxypropyl trimethyl ammonium chloride solution was dropped into the front mixture with stirring at 80 °C. After 10 h, the pH was adjusted to 7 at room temperature prior to filtering the mixture, and then the mixture was poured into substantial anhydrous ethanol. The leached compounds were rinsed thoroughly with 85% ethanol, dewatered with anhydrous ethanol, and dried in vacuo.

#### 3.3.2. Synthesis of Hydroxypropyltrimethyl Ammonium Chitosan Halogenated Acetates

HACC was dissolved in 20% sodium halogenated acetate for purpose of replacing the chloride ions with haloacetic ions. The solution was dialyzed with distilled water for 48 h, and quaternary ammonium salts of chitosan bearing halogenated acetate including hydroxypropyltrimethyl ammonium chitosan bromacetate (HACBA), hydroxypropyltrimethyl ammonium chitosan chloroacetate (HACCA), hydroxypropyltrimethyl ammonium chitosan dichloroacetate (HACDCA), hydroxypropyltrimethyl ammonium chitosan trichloroacetate (HACTCA), hydroxypropyltrimethyl ammonium chitosan difluoroacetate (HACDFA), and hydroxypropyltrimethyl ammonium chitosan trifluoroacetate (HACTFA) were obtained after drying in vacuo (Scheme 1).

### 3.4. Antifungal Assays

Antifungal assay against *C. lagenarium*, *F. oxysporum*, *B. cinerea*, and *P. asparagi* was performed by following the plate growth rate method [34]. Firstly, the samples (chitosan and chitosan derivatives) were dissolved in distilled water at a concentration of 6.0 mg/mL. Then, in a sterile environment, 2.0 mL, 1.0 mL, and 0.2 mL sample solutions were shaken into the fungus culture medium and poured into a disposable culture dish respectively to solidify. Therefore, the final concentrations of the samples were 1.0 mg/mL, 0.5 mg/mL, and 0.1 mg/mL, respectively. Finally, the fungi mycelium of 5.0 mm diameter was inoculated into the solidified solid medium center with tweezers and cultured at 28 °C for three days. The unsampled deionized water was used as a blank control until the mycelia of the blank group grew to the edge of the dish. Next, the diameter of mycelium growth area was measured and the average value was taken. The inhibitory index was calculated as follows:

(4) $$\mathrm{Inhibitory}\;\mathrm{index}\;(\%)\;=\;(1-D_{a}/D_{b})*100\;$$

where *D*_a_ is the diameter of the growth zone in the test plates, and *D*_b_ is the diameter of the growth zone in the control plate.

### 3.5. Statistical Analysis

The results of the antifungal activities were processed by Excel (2007, Microsoft Corporation, Redmond, WA, USA), Origin (8.0, OriginLab, Northampton, MA, USA), and the statistical software SPSS (19.0, SPSS, Chicago, IL, USA). Duncan’s methods by SPSS were used to evaluate the differences in the inhibitory index in the antifungal tests. The data were analyzed by an analysis of variance. When *p* < 0.05, the results were considered statistically significant.

## 4. Conclusions

In this paper, hydroxypropyltrimethyl ammonium chitosan halogenated acetates were successfully synthesized from six different haloacetic acids and hydroxypropyltrimethyl ammonium chloride chitosan with high substitution degree. Through testing these antifungal activities against *C. lagenarium*, *F. oxysporum*, *B. cinerea*, and *P. asparagi*, we found that six different chitosan derivatives had better antifungal activities than chitosan and hydroxypropyltrimethyl ammonium chloride chitosan (HACC). In particular, the inhibitory activity decreased in the order: HACTFA > HACDFA > HACTCA > HACDCA > HACCA > HACBA > HACC > chitosan, which was consistent with the electron-withdrawing property of different halogenated acetate. In summary, halogen groups were introduced into the synthesized chitosan derivatives which contributed a lot to the antifungal action, and the magnitude of antifungal activity depended on the electronegativity of substituents. Meanwhile, considering the obvious toxicity of many halogenated fungicides, the sustained release action of chitosan could solve this problem to a certain extent, and it would largely remit the problem of environmental issue. In short, this paper provided a new way of preparing chitosan derivatives with excellent antifungal activity, which has the potential of becoming alternatives to some harmful pesticides for disease control.

## Figures and Tables

**Table 1:** Yields and the degrees of substitution of chitosan derivatives.

Componds	Yields (%)	Elemental Analyses (%)			Degrees of Substitution (%)
		C	N	C/N	
CS	-	41.47	7.56	5.48	-
HACC	65.27	39.71	7.41	5.35	67.71
HACCA	69.43	38.10	6.27	6.05	67.31
HACDCA	50.86	38.86	6.63	5.86	56.23
HACTCA	64.85	37.22	6.07	6.13	71.97
HACDFA	58.97	40.86	7.13	5.73	48.64
HACTFA	67.56	38.27	6.25	6.12	71.39
HACBA	65.84	40.64	6.70	6.06	67.89

## Supplementary Materials

The following are available online at http://www.mdpi.com/1660-3397/16/9/315/s1, Table S1: The degrees of substitution (DS) of chitosan derivatives.
