# Supplementary material for: Synthesis, Characterization, and Antifungal Property of Hydroxypropyltrimethyl Ammonium Chitosan Halogenated Acetates
Source: Mar Drugs. 2018 Sep 5;16(9):315. doi: 10.3390/md16090315 (PMC6165101; doi:10.3390/md16090315)
Supplement: Supplementary file 1 [file marinedrugs-16-00315-s001.pdf]

marine drugs

Supporting information

Synthesis, characterization, and antifungal property of hydroxypropyltrimethyl ammonium chitosan halogenated acetates

Yingqi Mi [1,2], Wenqiang Tan [1,2], Jingjing Zhang [1,2], Lijie Wei [1,2], Yuan Chen [1,2], Qing Li [1], Fang Dong [1,*], Zhanyong Guo [1,2,*]

[1] Key Laboratory of Coastal Biology and Bioresource Utilization, Yantai Institute of Coastal Zone Research, Chinese Academy of Sciences, Yantai 264003, China
[2] University of Chinese Academy of Sciences, Beijing 100049, China
* Correspondence: E-mail address: zhanyongguo@hotmail.com(Z.G.); E-mail address: fdong@yic.ac.cn(F.D.); Tel.:+86-535-2109171; fax:+86-535-2109000(Z.G.); Tel.:+86-535-2109165; fax:+86-535-2109000(F.D.)

Table S1. The degrees of substitution (DS) of chitosan derivatives.

**Componds**	HACC	HACCA	HACDCA	HACDFA	HACBA
**DS(%)**	71.30	66.00	61.00	48.00	68.00
